# No difference between using short and long intervals for distributed proficiency-based laparoscopy simulator training: a randomized trial

**DOI:** 10.1007/s00464-023-10522-y

**Published:** 2023-11-22

**Authors:** Diana Hai Yen Tang, Theresa Bruun Østdal, Anishan Vamadevan, Lars Konge, Kim Houlind, Morten Stadeager, Flemming Bjerrum

**Affiliations:** 1 Copenhagen Academy for Medical Education and Simulation, Centre for HR and Education, The Capital Region, Ryesgade 53B, 2100 Copenhagen, Denmark; 2 The Faculty of Health Sciences, University of Southern Denmark, Odense, Denmark; 3 Faculty of Health and Medical Sciences, University of Copenhagen, Copenhagen, Denmark; 4 Department of Vascular Surgery, Hospital Lillebaelt, University of Southern Denmark, Odense, Denmark; 5 Department of Surgery, Hvidovre Hospital, Copenhagen, Denmark; 6 Department of Gastrointestinal and Hepatic Diseases, Copenhagen University Hospital - Herlev and Gentofte, Herlev, Denmark

**Keywords:** Laparoscopy, Simulation, Training, Distributed, Spaced, Massed, Proficiency

## Abstract

**Background:**

Simulation-based training is increasingly used to acquire basic laparoscopic skills. Multiple factors can influence training, e.g., distributed practice is superior to massed practice in terms of efficiency. However, the optimal interval between training sessions is unclear. The objective of this trial was to investigate if shorter intervals between sessions are more efficient than longer intervals during proficiency-based laparoscopy simulator training.

**Methods:**

A randomized simulation-based trial where medical students (*n* = 39) were randomized to proficiency-based training with either 1–2 days (intervention group) or 6–8 days (control group) between training sessions. Both groups practiced a series of basic tasks and a procedural module until proficiency level on the LapSim® simulator. Both groups were given instructor feedback upon request. After reaching proficiency, participants were invited back for a retention test 3–5 weeks later and practiced the same tasks to proficiency again.

**Results:**

The mean time to reach proficiency during training was 291 (SD 89) and 299 (SD 89) min in the intervention and control group, respectively (*p* = 0.81). During the retention test, the mean time to reach proficiency was 94 (SD 53) and 96 (SD 39) minutes in the intervention and control groups, respectively (*p* = 0.91).

**Conclusion:**

We found no difference whether practicing with shorter intervals or longer intervals between training sessions when examining time to proficiency or retention.

**Supplementary Information:**

The online version contains supplementary material available at 10.1007/s00464-023-10522-y.

Surgical training is facing the constraints of work-hour limitations, demands for efficiency, and increased concerns regarding patient safety. Hence, much effort has been focused on developing efficient and effective training strategies for surgical residents, such as using simulation-based training [1].

Simulation-based training for surgical residents can shorten the learning curve and improve technical skills, thus ensuring basic competencies before operating on patients. Several studies have shown that technical skills learned in a simulated environment are transferable to the operating room [2–4]. For simulation-based training to be efficient it must be based on the principles of best practice, like proficiency-based training, employing instructor feedback, and using distributed practice [5].

Distributed practice refers to two or more learning opportunities that are spaced apart or distributed over time. This produces better learning than the same opportunities that occur in massed practice, and several theories have been proposed to account for the spacing effect. Learning opportunities that are spaced apart in time are more likely to receive a learner’s full attention, ultimately leading to better learning outcomes. More recent evidence suggests that spacing learning opportunities across different days may benefit memory due to sleep-dependent neural consolidation processes [6]. Post-training sleep is believed to be beneficial for memory as well [7, 8]. Neurophysiological studies in rodents suggest that this is due to an iterative “reactivation” of memory networks during sleep, strengthening synaptic connections and facilitating memory consolidation. The observation that patterns of neural activity representing recent experiences are “reactivated” in the sleeping brains of rodents suggests that sleep’s effect on human memory might be attributed to a repeated “replay” of experience during sleep allowing cortical memory networks to be strengthened and integrated [9, 10].

Distributed training has also been shown to be beneficial for simulation-based training of surgical skills [6], however, the optimal frequency for practicing remains unclear [6]. In another study conducted by De Win et al. it was discovered that, following a longer inter-training interval, the trainee most likely needs more time to reactivate the earlier learned skills and will benefit less from the given training session [11].

The objective of this trial was to investigate if training using shorter intervals (1–2 days) was superior to longer intervals (6–8 days) between training sessions when using proficiency-based training.

## Materials and methods

The trial was a single-center randomized superiority trial following the CONSORT statement [12]. The trial was submitted to The Regional Ethics Committee, which found that no ethical approval was necessary (ID: 22028642). The trial was registered at ClinicalTrials.gov (ID: NCT05834504).

### Setting

The data was collected at the simulation center at Copenhagen Academy for Medical Education and Simulation (CAMES), Copenhagen, Denmark [13].

### Participants

Participants were eligible for inclusion if they were medical students. Participants were recruited from The University of Copenhagen. They were invited by e-mail and received written and verbal information before filling out the informed consent forms.

Exclusion criteria were I: Previous experience with laparoscopic surgery, II: Having participated in prior studies or similar involving laparoscopic training, III: Performing laparoscopy surgery between the intervention and the retention test 3–5 weeks after, IV: No informed consent, V: Did not speak Danish on a conversational level, VI: Any disability or injury regarding eyesight and mobility, VII: Did practice laparoscopic skills between the intervention and retention.

### Randomization

Before randomization, all participants were given a unique trial identification number and received individual information on the proper use of the simulator and instrument handling by one of the three investigators (DT, TØ, or AV).

The participants were then randomized to either the intervention group which practiced with 1–2 days between training sessions or the control group which practiced with 6–8 days between sessions.

The randomization was done using an online web-based system, Sealed Envelope (London, United Kingdom). A 1:1 randomization with randomly permuted blocks was used. The allocation sequence was generated by computer using varying block sizes of four and six. This was kept concealed from the investigators throughout the trial. Participants were stratified according to sex (man/woman).

### Intervention

All participants practiced four basic laparoscopic skills modules (Grasping, Lifting and grasping, Fine dissection, Cutting) and a procedural module (laparoscopic salpingectomy due to a bleeding ectopic pregnancy) in a self-directed manner. The participants reached the proficiency level for each module when they passed it twice within five consecutive attempts [14]. Practice sessions were booked via e-mail, and each session had a maximum time limit of two hours. An investigator was present at all practice sessions and participants had the opportunity to receive instructor feedback upon request. Instructor feedback consisted of technical questions about the equipment/software, help to understand the module, and suggestions on how to improve performance.

Upon reaching proficiency, participants were invited back after a break of 3–5 weeks to complete a retention test where they had to reach proficiency again. The amount of instructor feedback requested during the intervention and the retention test was registered by the investigators.

### Materials and equipment

**Fig. 1:**
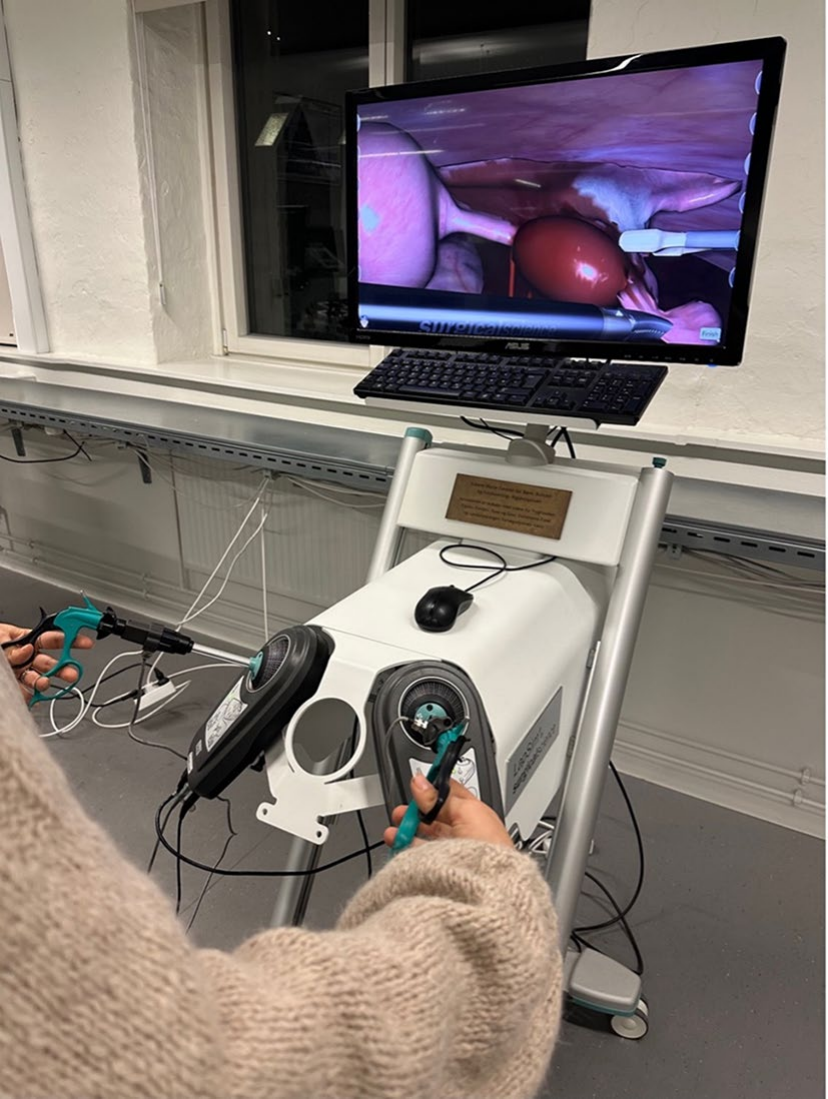
The Lapsim® simulator used in the study

We used three identical Lapsim® non-haptic virtual reality simulators (Surgical Science, Gothenburg, Sweden) connected to a server storing all participant data electronically (Fig. 1). The simulator consists of a physical user interface with laparoscopic instruments connected to a computer. The LapSim® software (version 2016.17) generates a virtual operating field, which can be viewed on a computer screen. The participants could interact with the virtual operating field and perform basic skills through the user interface.

### Outcome measures

The primary outcome was the total effective training time (minutes) to reach the proficiency level for all five simulator tasks. The secondary outcome was the total effective training time (minutes) during the retention test. Exploratory outcomes were the total time of instructor feedback needed (seconds) during the intervention and the retention test.

### Sample size calculation

Based on data from a previous trial, it was assumed the control group would require a mean of 320 min to reach proficiency level [15]. We chose a minimal relevant difference of 90 min. The standard deviation was assumed to be 80 min in both groups.

Based on these assumptions and using a power of 90% and a significance level of 5%, a sample size of a minimum of 34 participants (minimum of 17 in each group) was required [16].

### Statistical analysis

Intergroup comparisons for the primary and secondary outcomes during the intervention and the retention test were done using an independent samples *t* test, whereas the amount of requested instructor feedback was analyzed using a Mann–Whitney test. The effect of the intervention over time was analyzed using two-way repeated measurement ANOVA. The analysis of our data was done using SPSS® version 28.0 (IBM, Armonk, NY, USA).

## Results

**Fig. 2:**
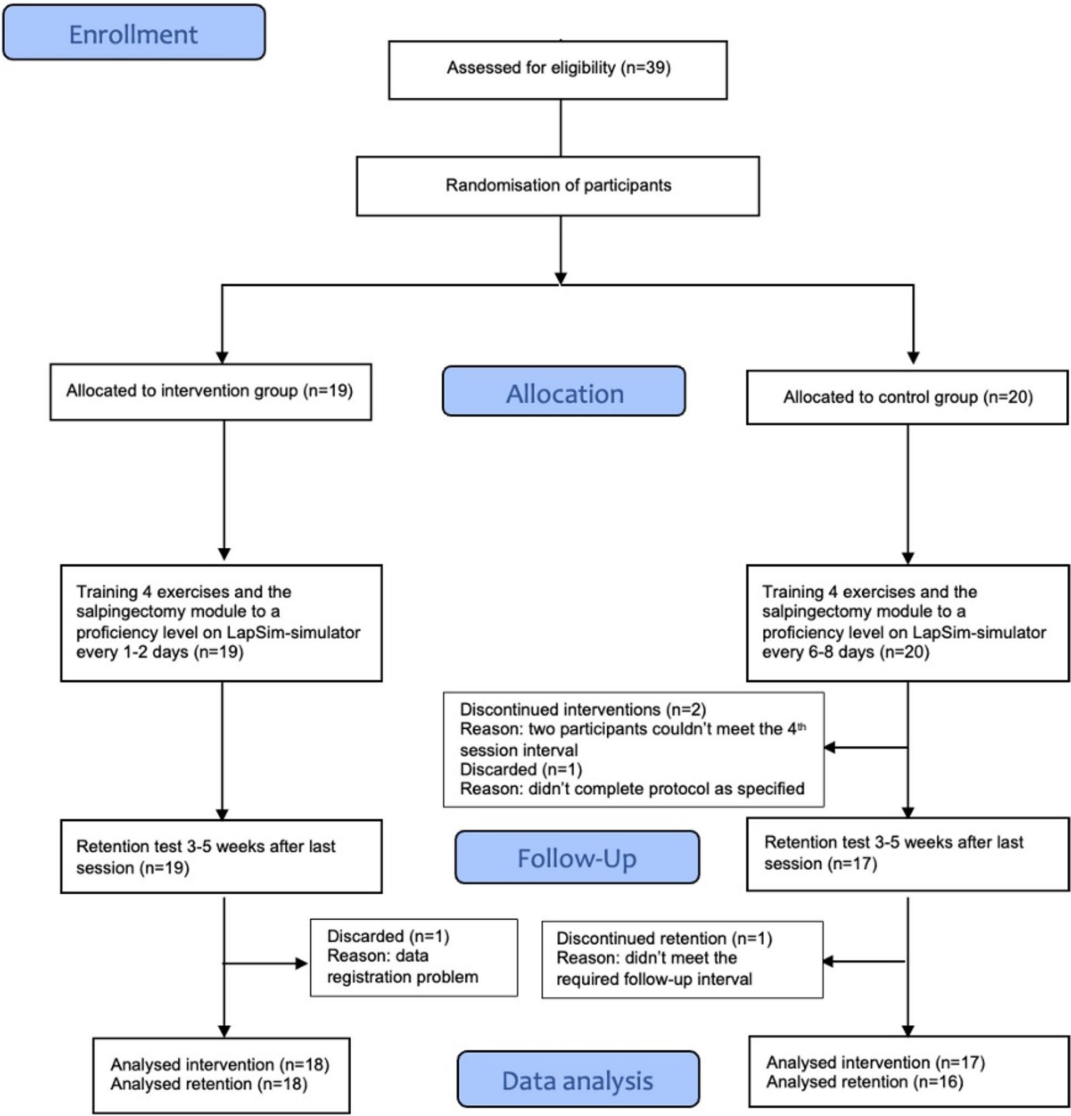
Trial flowchart according to the CONSORT flow diagram [12] for randomized trials

We included a total of 39 participants, of which 35 participants completed both the intervention and the retention test. Participant baseline characteristics are presented in Table 1 and the participant flowchart, Fig. 2. The participants were predominantly senior medical students, meaning they had completed their basic subjects and were starting the clinical courses. The intervention and control groups were comparable in this regard.

**Table 1:** Baseline characteristics for participants who completed the intervention

Groups	Intervention (*n* = 8)	Control (*n* = 17)
Sex (number of men/women)	3/15	7/10
Age [median (range)]	22.5 (20–28)	23 (20–25)
Dexterity (number of right-/left-handed	17/1	14/3

**Fig. 3:**
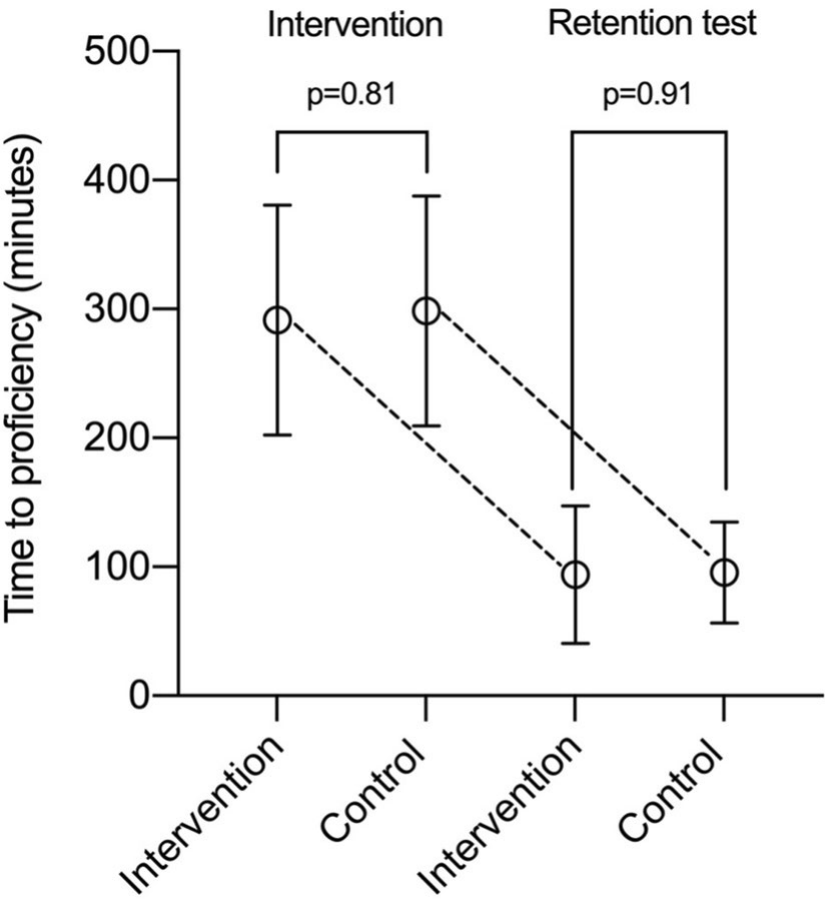
Intervalplot showing time to proficiency during the intervention and the retention test

The mean time to proficiency was 291 min (95% CI [113;469], SD 89) and 299 min (95% CI [121;477], SD 89), respectively, for the intervention and control group (*p* = 0.81). In the retention test the mean time to proficiency was 94 min (95% CI [− 13;201, SD 53) and 96 min (95% CI [18;174], SD 39), respectively, in the intervention and control group (*p* = 0.91) (Fig. 3).

Both groups reached proficiency significantly faster at the retention test compared to the initial training (*p* < 0.001). There was no effect of the intervention (*p* = 0.66) on the level of retention, meaning both groups showed the same level of retention.

There was no difference in the need for instructor-based feedback between the groups during the intervention. They used, respectively, 288 (IQR 76–467) and 236 (IQR 12–450) seconds in the intervention and control group (*p* = 0.52). For the retention test, the median time was 0 (IQR 0–0) in both groups (*p* = 0.37). During the intervention, two out of 18 participants in the intervention group and three out of 17 participants in the control group did not request any instructor feedback. During the retention test, 14 out of 18 participants from the intervention group and 12 out of 17 participants from the control group did not request instructor feedback.

## Discussion

We found that training with intervals of 1–2 days was not superior to intervals of 6–8 days in a proficiency-based laparoscopy simulator training program and that the two training schedules gave comparable results. Previous studies have shown that laparoscopic skills are acquired more efficiently in a distributed manner compared with massed practice [11, 17]. But exactly how training should be distributed, is still up for debate. A study conducted by Stefanidis et al. analyzed performance data from three randomized controlled trials following a similar proficiency-based simulator curriculum in laparoscopic suturing on the Fundamentals of Laparoscopic Surgery model. They found no significant association between inter-training interval (1–2 days, 3–4 days, 5–7 days, 8–14 days, and > 14 days) and change in performance, although shorter duration per session was associated with improved skill acquisition [18]. In our trial, training sessions were limited to two hours. The advantage of these shorter training sessions is that it reduces the likelihood of trainees becoming overly fatigued or bored during practice as mentioned in a previous study by Kahol et al. [19].

In contrast, a study conducted by Spruit et al. showed that spacing laparoscopic training over three consecutive days or weeks was superior to massed training. Even when the massed training contained breaks, breaks with sleep opportunities between sessions enhanced the performance compared to training with shorter breaks and massed training [17].

Other studies show that surgical training is most efficient when scheduled across multiple shorter time intervals, preferably with several non-training days between training sessions [20, 21]. A possible explanation for this is that getting overnight sleep between sessions enhances consolidation of the newly acquired skills because consolidation occurs in the brain when a person is disengaged from the trained activity [22, 23]. The theoretical downside of spending longer time between sessions is that retention may be reduced after longer periods without training. A study by Güldner et al. found that an inter-training interval of one week might be too long, especially for advanced exercises [24]. As mentioned earlier it was discovered that the trainee most likely needs more time to reactivate the earlier learned skills and will benefit less from the given training session following a longer inter-training interval [11]. These mentioned negative effects of the inter-training interval of one week were not found in our trial.

Our findings indicate that the most important issue in distributed practice is that there is at least one night of sleep between training sessions. This means that training programs should accommodate this, but also be flexible to fit with the work schedule of the surgical trainees. Our findings are also relevant for the organization of time-limited skills courses, which typically use massed practice on one or more days. If possible, training should be spread out over several days in shorter sessions, instead of doing it all on the same day, as this is more efficient for skills acquisition and reduces the risk of overload as earlier studies show [6, 11, 17].

The distribution of training did not impact the amount of instructor feedback needed in the intervention and during the retention test. This is in line with our main findings that the training schedules did not influence the effectiveness of the training. That there was no difference in the need for instructor feedback supports that there is a limited deterioration of skills during intervals of up to 8 days.

A strength of our trial was that we used a proficiency-based design over a time- or repetition-based training, which only focuses on the initial part of the learning curve. Additionally, our intervention was structured similarly to an actual simulation-based training program, meaning our findings can easily be applied to training programs. With the power of 90%, recruitment of more participants would not change the result of our study.

It is a limitation that we only compared two training schedules, and had we included an additional group with an even longer interval between sessions we could theoretically have gotten different results. There is also a small risk of bias due to the 2-week interval where the retention test could be done and some could have practiced in the beginning and the end of the interval. However, a study by Bjerrum et al. found that there was still significant retention of skills acquired after proficiency-based laparoscopy simulator training after a period of 6 months without training, showing retention of skills over long periods of time, making this less likely [25].

Furthermore, neither the investigators nor the participants were blinded to the intervention; we used medical students as participants and not doctors who are the actual target group of the laparoscopic training program, but the medical students were novices in laparoscopy similar to new residents and we believe the findings can be applied to this group. Finally, we did not examine transfer to a clinical setting: therefore, we cannot conclude anything about the impact of training intervals on actual clinical performance.

In conclusion, we did not find any benefit of shorter intervals compared with longer intervals between training sessions in a proficiency-based laparoscopy simulator training program.

### Supplementary Information

Below is the link to the electronic supplementary material.

Supplementary file1 (DOCX 16 kb)
