# Supplementary material for: No difference between using short and long intervals for distributed proficiency-based laparoscopy simulator training: a randomized trial
Source: Surg Endosc. 2023 Nov 22;38(1):300–5. doi: 10.1007/s00464-023-10522-y (PMC10776690; doi:10.1007/s00464-023-10522-y)
Supplement: Supplementary file 1 — Supplementary file1 (DOCX 16 kb) [file 464_2023_10522_MOESM1_ESM.docx]

**Supplementary: Proficiency settings for the basic Skills and the procedural Module**

Basic skill 1: Grasping

| **Parameter** | **Requirements for proficiency level** |
| --- | --- |
| Left instrument time (s) | < 45 |
| Left instrument path length (m) | < 2 |
| Left instrument angular path (degrees) | < 300 |
| Right instrument time (s) | < 45 |
| Right instrument path length (m) | < 2 |
| Right instrument angular path (degrees) | < 300 |
| Tissue damage (frequency) | < 3 |
| Maximum damage (mm) | < 5 |

Basic skill 2: Lifting and grasping

| **Parameter** | **Requirements for proficiency level** |
| --- | --- |
| Total time (s) | < 120 |
| Left instrument misses (%) | < 60 |
| Left instrument path length (m) | < 3.2 |
| Left instrument angular path (degrees) | < 600 |
| Right instrument misses (%) | < 60 |
| Right instrument path length (m) | < 3.2 |
| Right instrument angular path (degrees) | < 600 |
| Tissue damage (frequency) | < 5 |
| Maximum damage (mm) | < 15 |
| Grasper collided with left box (frequency) | < 10 |
| Left box lifted (frequency) | < 15 |
| Grasper collided with right box (frequency) | < 10 |
| Right box lifted (frequency) | < 15 |

Basic skills 3: Fine dissection

| **Parameter** | **Requirements for proficiency level** |
| --- | --- |
| Total time (s) | < 150 |
| Ripped or burned blood vessels | < 0 |
| Energy damaged on blood vessels (%) | < 20 |
| Ripped small vessels (%) | < 25 |
| Burned small vessels (%) | < 25 |
| Grasper path length (m) | < 0.5 |
| Grasper angular path (degrees) | <120 |
| Grasper outside view (frequency) | <2 |
| Grasper outside view (s) | <4 |
| Cutter path length (m) | <0,8 |
| Cutter angular path (degrees) | <200 |
| Cutter outside view (frequency) | <2 |
| Cutter outside view (s) | <4 |

Basic skill 4: Cutting

| **Parameter** | **Requirement for proficiency level** |
| --- | --- |
| Total time (s) | < 200 |
| Rip failure (%) | < 25 |
| Drop failure (%) | < 25 |
| Cutter path length (m) | < 2 |
| Cutter angular path (degrees) | < 400 |
| Grasper path length (m) | < 1.8 |
| Grasper angular path (degrees) | < 400 |
| Max stretch damage (%) | < 100 |
| Tissue damage (frequency) | < 10 |
| Maximum damage (mm) | < 25 |

Procedural module: Ectopic Pregnancy

Parameters for the procedure: salpingectomy on the Lapsim® virtual reality simulator. *To reach the proficiency level, all the proficiency level requirements must be fulfilled by using the correct operation technique.*

| **Parameters** | **Requirements for proficiency level** |
| --- | --- |
| Total time (s) | <280 |
| Left instrument path length (m) | <2 |
| Left instrument angular path (degrees) | <350 |
| Right instrument path length (m) | <3 |
| Right instrument angular path (degrees) | <450 |
| Blood loss (ml) | <180 |
| Pool of blood (ml) | < 10 |
| Ovary Diathermy damage (s) | < 3 |
| Tube Cut:  Uterus distance (mm) | < 4 |
| Removed dissected tissue (Yes/No) | Yes |
| Bleeding vessel cut (Yes/No) | No |
